# In Vivo and In Vitro Antioxidant Activity of Less Polar Fractions of *Dasycladus vermicularis* (Scopoli) Krasser 1898 and the Chemical Composition of Fractions and Macroalga Volatilome

**DOI:** 10.3390/ph15060743

**Published:** 2022-06-13

**Authors:** Sanja Radman, Ana-Marija Cikoš, Sanja Babić, Lara Čižmek, Rozelindra Čož-Rakovac, Stela Jokić, Igor Jerković

**Affiliations:** 1 Department of Organic Chemistry, Faculty of Chemistry and Technology, University of Split, Ruđera Boškovića 35, 21000 Split, Croatia; sradman@ktf-split.hr; 2 Department of Process Engineering, Faculty of Food Technology, Josip Juraj Strossmayer University of Osijek, Franje Kuhača 18, 31000 Osijek, Croatia; acikos@ptfos.hr (A.-M.C.); sjokic@ptfos.hr (S.J.); 3 Laboratory for Aquaculture Biotechnology, Division of Materials Chemistry, Ruđer Bošković Institute, Bijenička Cesta 54, 10000 Zagreb, Croatia; sanja.babic@irb.hr (S.B.); lara.cizmek@irb.hr (L.Č.); rozelindra.coz-rakovac@irb.hr (R.Č.-R.)

**Keywords:** benzaldehyde, 2-phenylbut-2-enal, pheophytin *a* and its derivatives, radical scavenging and antioxidant power, zebrafish model

## Abstract

The present research is a comprehensive investigation of *Dasycladus vermicularis* (Scopoli) Krasser 1898 from the Adriatic Sea (Croatia) regarding volatilome–volatile organic compounds (VOCs, mostly nonpolar compounds) and less polar nonvolatile compounds for the first time. Headspace solid-phase microextraction (HS-SPME) and hydrodistillation (HD) were used showing the great volatilome variability among fresh (HS-FrDV and HD-FrDV) and dried (HS-DrDV and HD-DrDV) samples after GC–MS analysis. Aromatic aldehydes were dominant in both fresh and air-dried HS samples with benzaldehyde as the most abundant in fresh samples and decreasing 2.7–3.7 times after drying together with 2-phenylbut-2-enal that was not present after drying. Aliphatic compounds (unsaturated hydrocarbons in HS-FrDV; saturated hydrocarbons in HS-DrDV) were also present. C_11_-hydrocarbons (dictyopterpene C’ and dictyopterpene D’) were detected in HS-FrDV. (*E*)-Phytol was the most dominant compound in HD-FrDV and HD-DrDV. Diterpene alcohols (cembra-4,7,11,15-tetraen-3-ol and (*Z*)-falcarinol) and sesquiterpene alcohol, cubenol, were dominant in HD-FrDV, and their abundance decreased after drying. C_13_-norisoprenoides (α-ionone and β-ionone) increased after drying. Aliphatic compounds were present in both HD-FrDV and HD-DrDV samples. The less polar nonvolatile compounds in the obtained fractions F3 and F4 were analysed and identified by UHPLC-ESI(+)-HRMS. Identified compounds belonged to a group of pigments (7 compounds), fatty acid derivatives (13 compounds), as well as steroids and terpenes (10 compounds). Porphyrin-based compounds (C_55_H_74_N_4_O_5–7_), xanthophylls, sphingolipid compounds, fatty acid amides, and phytosterols represented the majority of identified compounds. By implementing both in vitro and in vivo assays for antioxidant activity determination, F3 showed a higher activity than F4. Inhibitory concentrations (IC_50_) for F3 and F4 were 498.00 ± 0.01 µg/mL and 798.00 ± 0.81 µg/mL, respectively, while a 1.5-fold reduction in the ROS level was observed after pre-treatment of zebrafish larvae with 45 µg/mL of F3.

## 1. Introduction

The pharmaceutical industry is in a great rise at the global level, which has caused the necessity for new sources of pharmaceutical compounds such as the potential of marine algae. Cyanobacteria (blue-green algae) are well known for their production of antibiotics and active pharmaceutical compounds (API) [1,2]. Despite increasing interest, the economic utilisation of macroalgae for their antioxidant [3,4], anti-tumour [4], and antiviral properties [5] remains in its infancy. They contain complex structure compounds with a variety of biological activities, such as terpenes (antioxidant, anti-tumour, antifungal, etc.), alkaloids (antioxidant, anti-tumour, antiviral, anti-inflammatory, antibacterial, neuroprotective, etc.), and pigments (antioxidant, anti-tumour, anti-inflammatory, antidiabetic and antiobesity, neuroprotective, etc.) [6,7].

*Dasycladus vermicularis* (Scopoli) Krasser 1898 (fam. Dasycladaceae) is a green macroalga that inhabits rocky substrates of North Atlantic and Mediterranean littoral zones. To survive under increased UV exposure and temperatures, macroalgae developed antioxidant defence mechanisms that activate under the overproduction of reactive oxygen species (ROS), thus reducing/preventing the occurrence of oxidative stress [8]. Photobiological adaptation is strongly influenced by growth location, harvesting season, and environmental pressure, which can result in the variation in antioxidant molecules even among the same species. The Adriatic Sea is an extremely harsh environment characterised by its high salinity, relatively low depth, and oscillations in temperature, which might reflect in the occurrence of a variety of novel bioactive compounds with beneficial properties [9]. Currently available studies on *D. vermicularis* mostly focused on phenolic compounds with antioxidant and UV-radiation absorption activities such as coumarins and their sulfated metabolites such as 7-hydroxycoumarin-3,6-disulfate [9] and also isocoumarins [10]. There is a lack of data in the literature concerning volatile organic compounds (VOCs) analysis of *D. vermicularis*.

Our previous studies [10,11,12,13] showed that there is a great diversity of VOCs (mainly nonpolar compounds) when comparing fresh and air-dried samples of marine algae. This was the reason to analyse both fresh (FrDV) and air-dried (DrDV) samples to determine the VOCs variability of *D. vermicularis* using gas chromatography–mass spectrometry (GC–MS). In addition to the VOCs analysis, less polar nonvolatile compounds and their in vitro and in vivo antioxidative activities were investigated for the first time in this research. Therefore, full chemical profiles of volatile and nonvolatile polar compounds of *D. vermicularis* were investigated. The present research, as a continuation of our project Bioprospecting of the Adriatic Sea, has the following key goals: (a) identify and compare VOCs of FrDV and DrDV retrieved by both headspace solid-phase microextraction (HS-SPME) and hydrodistillation (HD) followed by gas chromatography–mass spectrometry analysis (GC–MS); (b) identify the composition of FdDV (freeze-dried *D. vermicularis*) less polar fractions using high-performance liquid chromatography–high-resolution mass spectrometry with electrospray ionisation (UHPLC-ESI–HRMS); (c) evaluate detailed antioxidant activity of the less polar fractions by four in vitro assays (reduction of radical cation ABTS^+^, 2,2-diphenyl-1-picryl-hydrazyl (DPPH) assay, Folin–Ciocalteu method, and ferric reducing antioxidant power (FRAP)); (d) determine the protective effect of the less polar fractions against hydrogen peroxide-induced oxidative stress in the zebrafish model along with the embryotoxicity assessment.

## 2. Results and Discussion

### 2.1. Headspace Composition

To analyse the headspace composition, solid-phase microextraction was used (HS-SPME). A more complementary headspace profile was attained using two fibres of different polarities: divinylbenzene/carboxene/polydimethylsiloxane (DVB/CAR/PDMS) and polydimethylsiloxane/divinylbenzene (PDMS/DVB). In the FrDV headspace (HS-FrDV), 90.99% of VOCs were identified in total with DVB/CAR/PDMS fibre and 91.74% with PDMS/DVB fibre. In the DrDV headspace (HS-DrDV) with both fibres, 100% of VOCs were identified. Aromatic compounds were dominant in both fresh and air-dried HS samples—55.25% (DVB/CAR/PDMS fibre) and 55.74% (PDMS/DVB fibre) in HS-FrDV and 77.44% (DVB/CAR/PDMS fibre) and 69.32% (PDMS/DVB fibre) in HS-DrDV. Benzaldehyde was the most abundant in fresh samples (26.07%; 27.34%) and decreased 2.7–3.7 times after the drying, and the second-most abundant compound was aromatic aldehyde 2-phenylbut-2-enal (24.20%; 21.72%), which could not be found in HS-DrDV (it probably evaporated). On the other hand, benzyl alcohol was the most abundant in air-dried samples (57.92%; 52.48%) (Table 1), as was found in the research of other algae after drying [11,12].

The second-most abundant group of compounds belonged to aliphatic compounds: unsaturated hydrocarbons in HS-FrDV (30.77%; 29.28%) and saturated hydrocarbons in HS-DrDV (16.43%; 22.85%). C_11_-hydrocarbons (well-known pheromones) dictyopterpene C’ (8.65%; 9.34%) and dictyopterpene D’ (7.78%; 7.12%) with the highest abundance in HS-FrDV decreased after the drying. Dictyopterene C’ could not be detected in HS-DrDV and the abundance of dictyopterpene D’ decreased 4.3–5.0 times after drying, which can be explained by the oxidative degradation [14,15,16,17]. In HS-DrDV, hexanal had the highest abundance (3.40%; 6.12%) among aliphatic compounds but could not be detected in HS-FrDV. This increment after air-drying was probably a consequence of fatty acids degradation.

### 2.2. Composition of the Volatiles Obtained by Hydrodistillation

In the hydrodistillate of FrDV (HD-FrDV) and of DrDV (HD-DrDV), 86.36% and 90.88% of TIC (total ion chromatogram) areas were identified altogether, respectively. Aliphatic compounds were dominant in both fresh (43.00%) and air-dried (61.42%) HD samples. In HD-FrDV, the majority of aliphatic compounds were hydrocarbons (both saturated and unsaturated), with the long-chain hydrocarbon docosane (7.69%) as the most abundant (Table 2). It decreased 23.6 times after the drying. The portion of unsaturated hydrocarbons increased 2.0 times after the drying with (*E*)-nonadec-9-ene (12.79%) as the most dominant. This could probably be the result of fatty acids’ decarboxylation, which follows its 4.4 times abundance decrement in HD-DrDV. The increment in saturated (2.4 times) and unsaturated alcohols (7.5 times) in HD-DrDV could be an indicator of the hydrocarbons’ oxidation during the drying. Among them, hexadecan-1-ol (10.37%) and (Z)-octadec-9-en-1-ol (8.13%) were the most abundant.

Diterpene alcohols (cembra-4,7,11,15-tetraen-3-ol and (Z)-falcarinol) as well as sesquiterpene alcohol, cubenol, were dominant among the group of terpenes (17.80%) in HD-FrDV. The abundance of terpenes dropped down 3.3 times in HD-DrDV.

The abundance of carotenoid degradation products, C_13_-norisoprenoides (α-ionone and β-ionone), increased 7.3 times after the drying mostly because of the increment in β-ionone, most probable due to carotenoids degradation.

### 2.3. Analysis of F3 and F4 Fractions Containing Less Polar Nonvolatile Compounds

**Figure 1:**
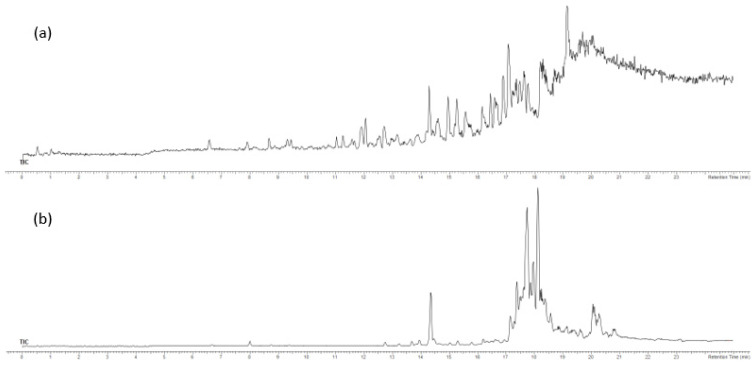
Total ion chromatograms (TIC) of the fractions (**a**) F3 and (**b**) F4.

**Figure 2:**
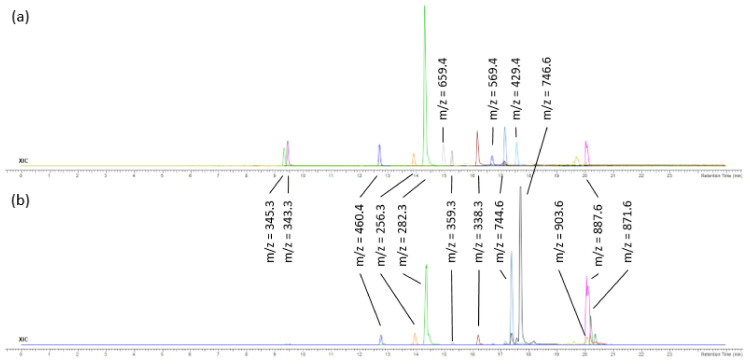
Extracted ion chromatograms (XIC) of the most abundant ions in the fractions (**a**) F3 and (**b**) F4.

The FdDV sample was extracted and fractionated (Section 3.6). F3 and F4 fractions containing less polar nonvolatile compounds were acquired and were analysed by high-performance liquid chromatography–high-resolution mass spectrometry with electrospray ionisation in positive mode (UHPLC-ESI(+)–HRMS). The major compounds according to signal intensity (peak area in counts) from the obtained extracted ion chromatograms (XIC) in positive ion mode were plausibly identified. The identification was based on the compounds’ proposed elemental compositions and MS/MS spectra (Table 3). Identified compounds belonged to the group of pigments (7 compounds), fatty acid derivatives (13 compounds), as well as steroids and terpenes (10 compounds). The TICs of the fractions F3 and F4 are shown in Figure 1 and the XICs of the most abundant ions in F3 and F4 are shown in Figure 2.

**Figure 3:**
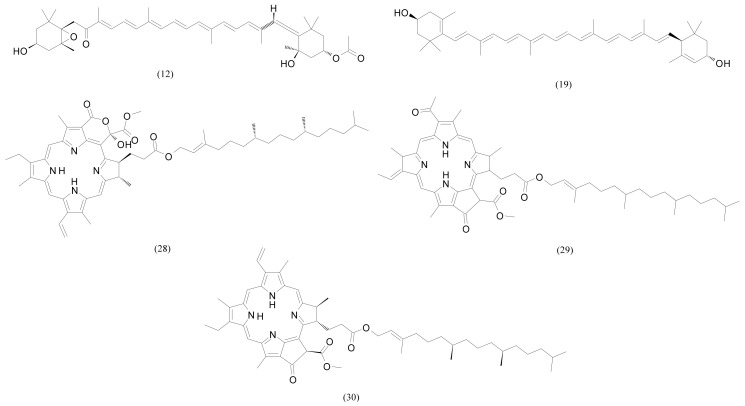
Molecular structures of the major pigments from Table 3, the numbers correspond to Table 3.

No chlorophyll was detected, but its derivatives, porphyrin-based compounds (C_55_H_74_N_4_O_5–7_), were abundant, especially in F4 (Figure 3). Among them, the main component was compound 29 (C_55_H_74_N_4_O_6_). Two other compounds were less polar, so they were much more abundant in F4. It is well known that the porphyrin ring is an essential part of the chlorophyll structure responsible for the antioxidant activity [18]. Antimutagenic and anti-inflammatory effects of pheophytin *a* were found in green alga *Ulva prolifera* (formerly known as *Enteromorpha prolifera*) [19] and its neuroprotective effect was found in brown alga *Sargassum fulvellum* [20]. Pheophorbide *a* was also detected but in a small amount, more in F3 than in F4. It has also shown antioxidant activity in green alga *Ulva prolifera* [21].

Xanthophylls, both present in green algae, zeaxanthin (or lutein; the isomers that could not be specified), and the one more characteristic for brown algae, fucoxanthin, were detected and identified (Figure 3) with a greater abundance in F3. In algae, xanthophylls play an important role to protect photosynthetic apparatus against damage induced by high light intensities. This photoprotective role directly purges ROS (reactive oxygen species) within the thylakoid membrane [22,23]. Similarly, they act as anti-tumour and anti-inflammatory agents and are utilised for the prevention of coronary syndromes, cardiovascular diseases, and they play roles in structural action in neural tissue [24]. Supercritical fluid extraction was used to extract zeaxanthin from green alga *Nannochloropsis oculata* [25] and lutein was extracted from green alga *Scenedesmus almeriensis* [26]. The ester of siphonaxanthin, another xanthophyll specific for green algae, siphonein, was detected in both fractions, more abundant in F4.

**Figure 4:**
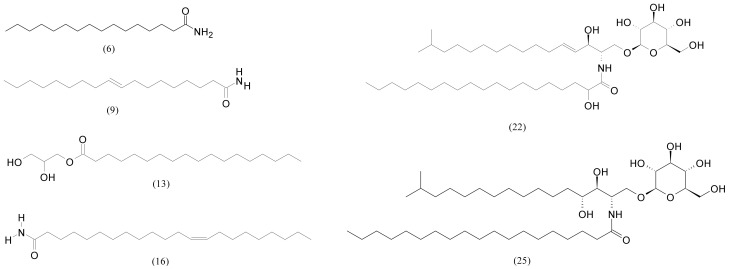
Molecular structures of the major fatty acid derivatives from Table 3, where the numbers correspond to Table 3.

Fatty acid derivatives in F3 and F4 originate from palmitic, stearic, oleic, and behenic acids (Table 3). Sphingolipid compounds 22 and 25 (Figure 4) were the most abundant of all detected components of F4, but were also present in F3. Sphingolipids as a part of macroalgae membranes play one of the crucial structural and physiological roles [27]. Recently, studies have shown that sphingolipid, together with associated enzymes and receptors, were involved in the inflammatory process and can provide effective drug targets for the treatment of pathological inflammation [28]. Three fatty acid amides were found: oleamide, erucamide, and palmitamide. They were more abundant in F4 even though oleamide was the most abundant of all detected compounds in F3. Oleamide is an endogenous bioactive signalling molecule found in both marine and terrestrial plants. Acting in various cell types consequently triggers different biological effects, and one of the most recognised ones is its sleep-inducing effect [29]. Isolated from green algae *Codium fragile*, oleamide also suppressed, among others, the secretion of TNF-α (tumour necrosis factor-α) and interleukins IL-1β (interleukin-1 beta) and IL-6 (interleukin 6), and prevented the translocation of NF-kappa B into the nucleus, which are main features in the anti-tumour process [30]. It was also found in *Ericaria* sp. [11]. Erucamide and palmitamide were previously identified in *Prymnesium parvum* [31] and *Ericaria* sp. [11]. Fatty acid glycerides were also present in both fractions.

**Figure 5:**
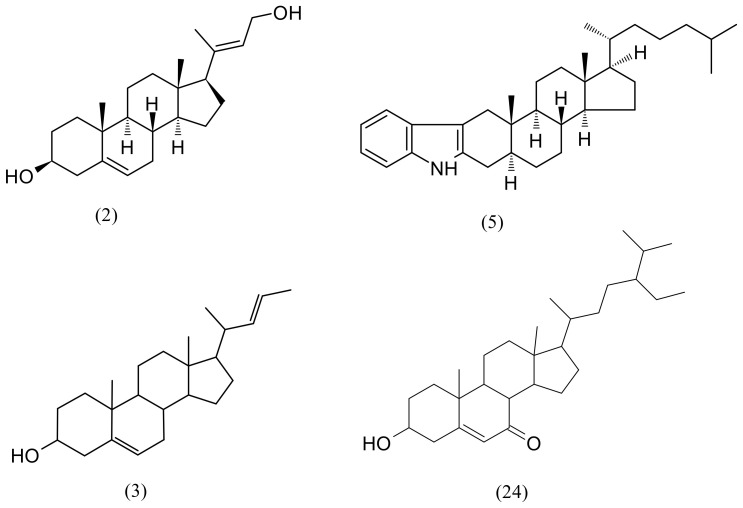
Molecular structures of the major compounds of steroids from Table 3, and the numbers correspond to Table 3.

Steroids and terpenes consisted of 10 identified compounds (Table 3). Four compounds were the major ones (Figure 5): sterols (compounds 2 and 3) and compound 24 (Table 3), which belong to the class of stigmastenes and derivatives that had a higher abundance in F3; and the steroid, compound 5 (Table 3) that was more present in F4. Phytosterols, a term referring to plant-derived sterols or stanols, are a group of naturally occurring compounds found in plant cell membranes [32]. Their main therapeutic function is reducing the cholesterol level [33], but they exhibit numerous other health benefits such as anti-diabetes, anti-atherosclerosis, anti-obesity, anti-Alzheimer’s, anti-tumour, and hepatoprotective [34,35]. Green algae have a relatively heterogeneous sterol composition [36]. Previous research found four sterols in *D. vermicularis*: cholesterol, 24-methylenecholesterol, brassicasterol, and clionasterol [37].

### 2.4. Antioxidant Activity of F3 and F4 Fractions In Vitro

**Figure 6:**
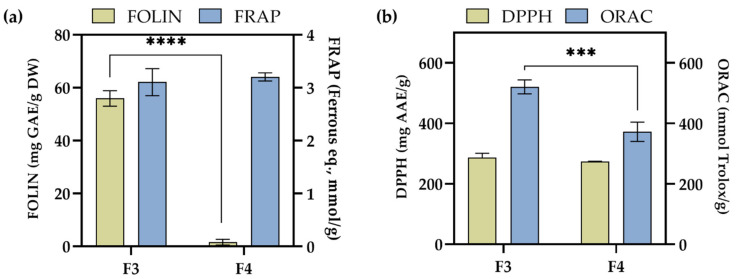
Scavenging radical activity of two fractions of *D. vermicularis* (F3 and F4) obtained using (**a**) Folin–Ciocalteu method and ferric reducing antioxidant power (FRAP), and (**b**) 2,2-diphenyl-1-picrylhydrazyl (DPPH) assay and oxygen radical absorbance capacity (ORAC) (mean ± SD; *n* = 4). An asterisk indicates a significant difference between F3 and F4 (*** *p* < 0.001; **** *p* < 0.0001).

To analyse the antioxidant potential of the obtained *D. vermicularis* fractions (F3 and F4), and to obtain better insight into their antioxidant behaviour, different assays were used. The results for four assays (Folin–Ciocalteu method, ferric reducing antioxidant power (FRAP), 2,2-diphenyl-1-picrylhydrazyl (DPPH), and oxygen radical absorbance capacity (ORAC)) are depicted in Figure 6. The results of Folin–Ciocalteu method showed significantly (*p* < 0.0001) different activity for F3 and F4 fractions, namely, a 50-fold higher activity was obtained for F3. As no polyphenols were found in these fractions (Table 3), this response for F3 could be assigned to the presence of xanthophylls fucoxanthin and zeaxanthin (or lutein) in higher amounts when compared to F4, as it is known that the pigments interact with Folin–Ciocalteu regent [38]. Contrarily, almost the same activity was obtained using the FRAP assay for both fractions, i.e., 3.1 ± 0.2 mmol/g for F3 and 3.2 ± 0.07 mmol/g ferrous equivalents for F4. The scavenging activity using DPPH assay when normalised per gram of the fraction revealed also almost the same activity for both fractions, i.e., for F3, it was 286.79 ± 14.09 mg AAE/g fraction, while for F4, it was 273.42 ± 1.29 mg AAE/g fraction. ORAC assay is based on a fluorescent signal from a probe that is quenched in the presence of reactive oxygen species (ROS). The results shown in Figure 6b revealed a 1.4-fold higher (*p* > 0.001) activity for F3 than F4.

**Figure 7:**
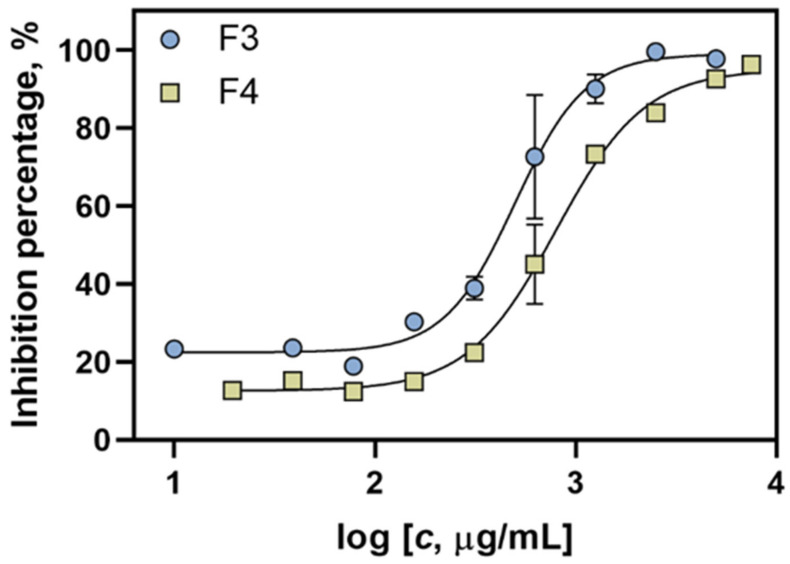
Concentration–inhibition response curves for F3 and F4 fractions from *D. vermicularis* used for the calculation of their antioxidant activity by using the reduction in the radical cation (ABTS assay).

Further on, the detailed reduction of radical cations by implementing ABTS assay was studied. Different concentrations of F3 and F4 fractions were prepared ranging from 0.005 to 5 mg/mL to obtain dose–response curves (Figure 7) and calculate IC_50_ values (Table 4). As can be seen, two *D. vermicularis* samples exhibited a similar activity, i.e., IC_50_ values were 0.498 and 0.798 mg/mL for F3 and F4 fractions, respectively. A lower IC_50_ value for F3 could be ascribed to higher amounts of xanthophylls found in F3. However, one should note that a similar antioxidant behaviour was observed for both F3 and F4 of *D. vermicularis*, which indicate that, although different compounds were identified in each fraction and in different amounts, a synergistic effect leading to a similar activity can be observed. Additionally, in F4, indole derivatives were found (for example, 1′H-5α-cholest-2-eno [3,2-b] indole) in a higher amount, which probably contributed to the antioxidant responses [39].

### 2.5. Antioxidant Activity of F3 and F4 Fractions In Vivo

**Figure 8:**
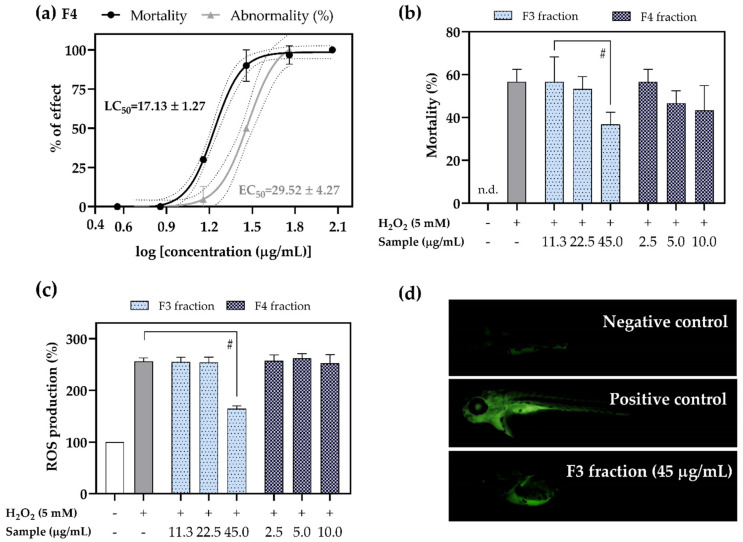
Acute toxicity of tested F3 and F4 fractions, along with their antioxidant potential in vivo: (**a**) concentration–response curves used for the calculations of *D. rerio* mortality and abnormality rates after 96 h of exposure to F4. Dotted lines represent 95% confidence intervals. Protective effect of F3 and F4 on the (**b**) survival and (**c**) ROS production hydrogen peroxide-stressed *D. rerio* embryos. (**d**) Qualitative analysis of ROS production in live specimens observed under fluorescent microscopy. The results are expressed as mean ± SD of three replicates; # denotes significant differences (*p* < 0.05); n.d. = not detected.

The antioxidant potential of F3 and F4 fractions was assessed in vivo in zebrafish *Danio rerio* exposed to H_2_O_2_-induced oxidative stress. F4 induced a concentration-dependent increase in mortality (half maximal lethal concentration (LC_50_) = 17.13 ± 1.27 µg/mL) and developmental abnormalities (half maximal effective concentration (EC_50_) = 29.52 ± 4.27 µg/mL), as presented in Figure 8a. Although no mortality was observed during the exposure to F3, the highest tested concentration (62 µg/mL) increased the incidence of developmental abnormalities (16.67 ± 11.55% of specimens; data not shown). When compared to F3 fraction, F4 fraction contained a higher amount of fatty acid amides such as oleamide, palmitamide, and erucamide that might be responsible for elevated toxicity. This was also the case in our recent study where *Ericaria crinita* and *Ericaria amentacea* fractions enriched with mentioned fatty acid amides negatively affected the survival of zebrafish embryos [11]. Considering the observed toxicity, the determination of in vivo antioxidant potential was decided to be conducted on 2.5, 5.0, and 10.0 µg/mL of F4 and 11.3, 22.5, and 45.0 µg/mL of F3. As shown in Figure 8b, the survival rate of the zebrafish larvae was significantly reduced upon exposure to H_2_O_2_. However, pre-treatment with 45 µg/mL of F3 significantly protected zebrafish from H_2_O_2_-induced mortality (survival rate increased by 35.3% compared to the treatment group on H_2_O_2_; *p* < 0.05). H_2_O_2_ increased ROS production in zebrafish up to 255.8 ± 6.99% compared to the control treatment group on artificial water (100%; Figure 8c), which revealed a high fluorescence of the positive control (Figure 8d). Following the treatment with 45 µg/mL of F3, the ROS levels reduced to 164 ± 6.2% (*p* < 0.05; Figure 8c,d).

These findings are in correlation with the results obtained with UHPLC-ESI(+)–HRMS analysis. Both fractions contain the compounds that have antioxidative properties such as porphyrin-based compounds [40] and xanthophylls [41], as well as sphingolipids [42]. Fucoxanthin and zeaxanthin/lutein (Table 3), molecules well-known for their antioxidant potential, have a greater abundance in F3, which might be the reason of the stronger antioxidant activity of F3 in relation to F4. Nevertheless, one should notice that the determination of the antioxidant potential of F4 in vivo was conducted within the lower concentration range (2.5–10.0 µg/mL) compared to F3 (11.3–45.0 µg/mL). Taken together, the obtained results indicate the pharmaceutical potential of *D. vermicularis*.

## 3. Materials and Methods

### 3.1. Chemicals

Water and acetonitrile, both containing 0.1% (*v*/*v*) formic acid, were both hypergrade (HPLC-MS LiChrosolv^®^) and purchased from Supelco Co. (Bellefonte, PA, USA).

The standards of gallic acid (>97.5%), TPTZ (2,4,6-tripyridyl-S-triazine, ≥98%), L-ascorbic acid (≥99%), DPPH (2,2-diphenyl-1-picrylhydrazyl), fluorescein, AAPH (2,2-azobis (2-methylpropionamidine) dihydrochloride, 97%), and ABTS (diammonium salt of 2,2-azino-bis(3-ethylbenzthiazolin-6-yl) sulfonic acid, >99.0%) were purchased from Sigma-Aldrich (St. Louis, MO, USA).

### 3.2. Alga Sample

*Dasycladus vermicularis* (Scopoli) Krasser 1898 was collected in July 2021 by a single-point collection from the Adriatic Sea (Uvala Jasenovo, Ravni Kotari) with the sampling geographical coordinates 44°17′01″ N; 15°12′27″ E. The sea depth was 3 m with the sea temperature at 25 °C. The macroalga was transferred to the laboratory in an air-tight plastic box containing both alga and seawater immediately after the collection. It was maintained at 4 °C in the dark for not more than 24 h until further analysis. A part of the collected *D. vermicularis* was air-dried and placed in the dark at room temperature for 10 days. Both fresh and air-dried samples were sliced into small pieces before further analysis. The identification of the collected alga was performed by marine biology experts Donat Ptericiolli and Dr Tatjana Bakran-Petricioli, professor at the Faculty of Science, University of Zagreb.

A part of *D. vermicularis* was freeze-dried for fractionation, as described in Section 3.6. Before the freeze-drying, the sample was washed 5 times in tap water and 2 times in deionised water and was sliced into 5–10 mm pieces. Sliced alga was frozen at −60 °C in an ultra-low-temperature freezer (CoolSafe PRO, Labogene, Denmark) for 24 h. The primary and secondary drying temperatures were −30 °C and 20 °C and freeze-drying was performed under a high vacuum (0.13–0.55 hPa) for 24 h.

### 3.3. Headspace Solid-Phase Microextraction (HS-SPME)

The excess seawater from the fresh sample was removed by placing a part of the sample between two layers of filter paper for a few minutes. HS-SPME was performed using an autosampler, PAL Auto Sampler System (PAL RSI 85, CTC Analytics AG, Zwingen, Switzerland), and two SPME fibres of different polarities. One fiber was covered with DVB/CAR/PDMS (divinylbenzene/carboxen/polydimethylsiloxane) and the other one with PDMS/DVB (poly-dimethylsiloxane/divinylbenzene). Both fibres were conditioned prior to the extraction according to the manufacturer and were purchased from Supelco Co. (Bellefonte, PA, USA). Prepared samples, with the mass of 1 g, were placed into HS-20 mL glass vials sealed with a polytetrafluorethylene (PTFE)/silicon septa stainless-steel cap. Equilibration of the sample was performed at 60 °C for 15 min and then it was extracted for 45 min. The temperature of the injector was set to 250 °C. Thermal desorption of the sample from the fibre was conducted directly to the GC column for 6 min. HS-SPME was performed in triplicate.

### 3.4. Hydrodistillation (HD)

The hydrodistillation (HD) procedure was obtained in a modified Clevenger apparatus for 2 h. Diethyl ether (J.T. Baker Inc., NJ, USA) and pentane (Fluka, Merck KGaA, Germany) were used as the solvent trap in a *v*/*v* ratio of 2:1 (1 mL). HD was performed separately on the prepared samples of fresh and air-dried *D. vermicularis*. The volatile oil dissolved and trapped in the solvent trap was carefully removed with a pipette avoiding taking the water part. It was then slowly concentrated by the gentle flow of nitrogen until the volume of 0.2 mL was reached. An amount of 2 µL of the sample was used for GC–MS analyses. HD was performed in triplicate.

### 3.5. Gas Chromatography Mass Spectrometry Analysis of VOCs

The GC–MS analyses of isolated VOCs were run on an Agilent Technologies (Palo Alto, CA, USA) gas chromatograph model 8890 tandem mass spectrometer detector model 5977E MSD (Agilent Technologies, Palo Alto, CA, USA). An HP-5MS capillary column with the dimensions of 30 m × 0.25 mm and 0.25 µm film thickness (Agilent Technologies, Palo Alto, CA, USA) was used for the VOCs separation. The injector temperature was set to 250 °C and the detector temperature to 300 °C. The oven temperature was set to 70 °C isothermal for 2 min. The temperature gradient was obtained by temperature increments from 70 to 200 °C at 3 °C/min. After reaching the temperature of 200 °C, it was held isothermally for 15 min. The flow rate of helium as a carrier gas was 1.0 mL/min and the split ratio was 1:50. The MSD (EI mode) was operated at 70 eV with the scanning mass range from 30 to 300 Amu. The identification of the compounds was achieved by comparing their retention indices (RI), which were defined relative to the retention times of *n*-alkanes (C_8_–C_25_), with those reported in the literature (National Institute of Standards and Technology) and their mass spectra with the spectra from NIST 17 (D-Gaithersburg) and Wiley 9 (Wiley, New York, NY, USA) mass spectral libraries. The percentage composition of the samples was calculated using the normalisation method without correction factors. The average component percentages in Table 1 and Table 2 were calculated from GC–MS analyses of three replicates.

### 3.6. Fractionation by Solid-Phase Extraction (SPE)

The freeze-dried *D. vermicularis* (FdDV) was extracted in methanol and dichloromethane (MeOH:DCM = 1:1, *v*/*v*; 10 mL/g solvent-solid ratio) with 5 min of sonication (ultrasound-bath Elma, Elmasonic P 70 H, Singen, Germany; 37 kHz/50 W) three times. The gained extract (with 6.0377 g/105.3 mg drug:extract ratio) was evaporated under a nitrogen flow (5.0, Messer, Croatia) and was then mixed with C18 powder (40–63 µm, Macherey-Nagel Polygoprep 60–50 C18, Fisher Scientific, Waltham, MA, USA). An SPE cartridge (C18 with particle size of 40 µm, column capacity of 6 mL, and bed weight of 1 g, Agilent Bond Elut, Waldbronn, Germany) was conditioned with MeOH and ultrapure water and then covered with dry extract. To obtain the fractions F1 to F4, the sample was eluted by applying the solvents of decreasing polarity [10]: F1 (H_2_O), F2 (H_2_O:MeOH = 1:1, *v*/*v*), F3 (MeOH), and F4 (MeOH:DCM = 1:1, *v*/*v*)). Less polar compounds were eluted in F3 and F4. The fractions were dried by SpeedVac (SPD1030, Thermo Scientific, Waltham, MA, USA) and stored at 4 °C in the dark before further analysis.

### 3.7. Ultra-High-Performance Liquid Chromatography–High-Resolution Mass Spectrometry (UHPLC-ESI–HRMS) of F3 and F4

The UHPLC-ESI–HRMS analyses were performed using an UHPLC ExionLC AD system (AB Sciex, Concord, ON, Canada), which was equipped with the ExionLC Controller, ExionLC AD Pump, ExionLC AD Degasser, ExionLC solvent delivery system, ExionLC AD Autosampler, and ExionLC AD Column oven tandem quadrupole-time-of-flight (Q-TOF) mass spectrometer TripleTOF 6600+ (AB Sciex, Concord, ON, Canada) having a Duospray ion source. The chromatographic separations were achieved on the analytical column Acquity UPLC BEH Phenyl-Hexyl with dimensions of 2.1 mm × 100 mm and a particle size of 1.7 µm (Waters, Milford, MA, USA). The aqueous mobile phase (A) was water containing 0.1% formic acid and the organic mobile phase (B) was acetonitrile with 0.1% formic acid. The mobile phase flow rate was continuously 0.4 mL/min and the oven temperature was 30 °C. The elution started isocratically for 0.6 min with 2% of B and, later, the gradient program was applied: 0.6–18.5 min (B linear gradient to 100%), 18.5–25 min (100% B). An amount of 4 µL of the sample was injected.

Positive electrospray ionisation (ESI+) was set. Collision-induced dissociation (CID) in information-dependent acquisition (IDA) mode was applied recording the MS/MS mass spectra. The precursor ions only with the signal intensities above the 200 cps threshold were recorded with the maximum number of 15 precursor ions simultaneously subjected to CID. The parameters set in the ion source were as follows: source temperature of 300 °C, nebulising gas (gas 1, air) pressure of 40 psi, curtain gas (nitrogen) pressure of 30 psi, heater gas (gas 2, air) pressure of 15 psi, and ESI capillary voltage of 5.5 kV. Mass spectra were recorded in the m/z range of 100–1000 (MS) with the declustering potential of 80 V and accumulation time of 100 ms. MS/MS data were recorded in the m/z range of 20–1000, and the collision energy of the collision gas (nitrogen) was 40 eV with a spread of 20 eV. The mass scale calibrations (in the MS and MS/MS modes) were performed prior to each run in an automatic regime using an ESI Positive Calibration Solution 5600 (AB Sciex, Concord, ON, Canada).

ACD/Spectrus Processor 2021.1.0. (ACD/Labs, Toronto, ON, Canada) was used for processing data. The accurate masses of the protonated molecules, their isotopic distributions, and the product ions m/z in MS/MS spectra were used for the compounds’ elemental compositions determination. Detected components were then identified based on their mass spectra and the elemental compositions linked to search in the Chemical Entities of Biological Interest (ChEBI) database. The choice among the suggested hits was based on MS/MS data matching.

### 3.8. Antioxidant Activity of Tested Fractions by In Vitro Assays

Antioxidant activity determination was performed using five different methods, namely the Folin–Ciocalteu method, ferric-reducing antioxidant power (FRAP), 2,2-diphenyl-1-picryl-hydrazyl (DPPH) assay, oxygen radical absorbance capacity (ORAC), and the reduction in the radical cation (ABTS) assays. All assays were carried out in triplicates in 96-well plates using a UV/Vis microplate reader (Infinite M200 PRO, TECAN, Switzerland), while all results were expressed as mean ± standard deviation (*n* = 4). A detailed description of all used methods in this research is described in our recently published manuscript [11].

### 3.9. Embryotoxicity Potential

Zebrafish maintenance and spawning have been described in our recent paper [43]. The zebrafish embryotoxicity test (OECD 236, 2013) was conducted to determine the negative potential of tested F3 and F4 fractions. A wide range of serial dilutions of F3 (115–3.59 µg/mL) and F4 (62–2.88 µg/mL) was tested, at the same time taking care that the concentration of the solvent (MeOH and DMSO) did not exceed 1%. As a negative control artificial water was used, while MeOH and DMSO (1%) were used as a solvent control. Upon 96 h of exposure to tested fractions, mortalities and abnormalities were inspected using an inverted microscope Olympus CKX41, equipped with a Leica EC3 digital camera and LAS EZ 3.2.0 digitising software. The concentrations of fractions that showed no negative impact on zebrafish embryonal development were further used.

### 3.10. Antioxidant Potential In Vivo

To determine the antioxidant potential of DAVE fractions in vivo, the protocol described in Jerković et al. (2021) was followed [12]. Briefly, zebrafish embryos were pre-treated with three concentrations of F3 (2.5, 5.0, and 10.0 µg/mL) and F4 (11.3, 22.5, and 45.0 µg/mL) for 2 h and stimulated with 5 mM of H_2_O_2_. At 96 hpf, survived specimens were counted. To detect intracellular ROS formation, larvae were stained with 10 µM of DCF-DA and incubated in the dark for 1 h. Stained larvae were inspected using the fluorescent microscope Olympus^®^ BX51 and images were taken using the digital camera DP70 and Microsoft^®^ AnalySIS Soft Imaging System Software 3.1. (Münster, Germany). The fluorescence intensity of images was quantified using ImageJ software.

### 3.11. Ethical Statement

Animal housing and spawning were performed in an aquaria unit approved by the Croatian Ministry of Agriculture and according to the Directive 2010/63/EU. The experiments were conducted on the nonprotected stages of zebrafish development (up to 96 hpf), which do not require permission by animal welfare commissions (Directive 2010/63/EU).

### 3.12. Statistical Analysis

GraphPad Prism software version 8 was used for statistical analysis and graph presentation. A one-way analysis of variance (ANOVA) and Tukey’s multiple comparisons were performed to examine the significant differences between the means. A Welch’s *t*-test was used to examine the significant difference between tested samples for each in vitro antioxidant method. Differences were considered significant at *p* < 0.05. Prior to LC_50_ and IC_50_ determination, the obtained values were subjected to logarithmic transformation.

## 4. Conclusions

Little data are available on *D. vermicularis* chemical composition in the literature, except for polyphenols and coumarins analysis, and the present research is a comprehensive investigation of *D. vermicularis* from the Adriatic Sea (Croatia) regarding VOCs and less polar nonvolatile compounds for the first time. VOCs from both fresh (FrDV) and air-dried (DrDV) samples were presented. Both HS-SPME and HD were used, showing a great variability among HS-FrDV and HD-FrDV, as well as among HS-DrDV and HD-DrDV. Utilising these two methods, VOCs of different polarities and volatility were isolated. There is clearly a noticeable difference in fresh (FrDV) and air-dried (DrDV) samples. Aromatic compounds were dominant in both fresh and air-dried HS samples with benzaldehyde and 2-phenylbut-2-enal as the most abundant in fresh samples. C_11_-hydrocarbons dictyopterpene C’ and dictyopterpene D’ were detected with great abundance in HS-FrDV. Aliphatic compounds were dominant in both HD-FrDV and HD-DrDV samples. In HD-FrDV, diterpene alcohols (cembra-4,7,11,15-tetraen-3-ol and (*Z*)-falcarinol) and sesquiterpene alcohol cubenol were dominant. Its amount decreased, but C_13_-norisoprenoides (α-ionone and β-ionone) increased during the air-drying process. The less polar compounds in F3 and F4 were analysed and identified by UHPLC-ESI(+)-HRMS. Identified compounds belonged to a group of pigments (7 compounds), fatty acid derivatives (13 compounds), as well as steroids and terpenes (10 compounds). Porphyrin-based compounds (C_55_H_74_N_4_O_5–7_), xanthophylls, sphingolipid compounds, fatty acid amides, and phytosterols represent the majority of identified compounds.

In vitro analysis of the antioxidant potential of less polar compounds from *D. vermicularis* by implementing five independent assays (Folin–Ciocalteu, FRAP, DPPH, ORAC, and ABTS) showed a high activity of both F3 and F4 fractions. F3 exerted a higher inhibition of reactive oxygen species than F4, which can be related to the presence of pigments, namely xanthophylls. When compared to the results obtained by in vivo analysis, F3 also showed protective effects against the H_2_O_2_-induced mortality of zebrafish embryos. The first results on *D. vermicularis* suggest that this green alga might be a potent source of natural antioxidants such as pigments, terpenes, and porphyrin-based compounds, and further research regarding different biological activities is in progress.

## Figures and Tables

**Table 1:** The VOCs from *D. vermicularis* isolated by headspace solid-phase microextraction (HS-SPME) and analysed by gas chromatography–mass spectrometry (GC–MS): (I—fresh *D. vermicularis* extracted by DVB/CAR/PDMS fibre, II—air-dried *D. vermicularis* extracted by DVB/CAR/PDMS fibre, III—fresh *D. vermicularis* extracted by PDMS/DVB fibre, IV—air-dried *D. vermicularis* extracted by PDMS/DVB fibre).

No.	Compounds	RI	Area (%) ± SD			
			I	II	III	IV
1	Pent-1-en-3-ol	<900	-	-	1.34 ± 0.26	-
2	Pentanal	<900	-	-	-	0.52 ± 0.10
3	Pentan-1-ol	<900	-	-	-	0.72 ± 0.05
4	(*Z*)-Pent-2-en-1-ol	<900	-	-	-	1.20 ± 0.10
5	Hexanal	<900	-	3.40 ± 0.82	-	6.12 ± 0.29
6	Nonane	900	-	-	2.73 ± 0.15	-
7	Heptanal	907	-	-	-	2.35 ± 0.14
8	Benzaldehyde	970	27.79 ± 1.29	9.82 ± 0.06	27.34 ± 1.20	7.30 ± 0.26
9	Phenol	984	1.50 ± 0.01	6.64 ± 0.07	2.86 ± 0.08	7.02 ± 0.18
10	Octan-3-one	991	2.96 ± 0.09	-	1.96 ± 0.023	-
11	6-Methylhept-5-en-2-one	995	-	-	2.13 ± 0.13	-
12	2-Pentylfuran	996	-	2.18 ± 0.29	-	2.52 ± 0.05
13	Octanal	1007	-	2.54 ± 0.22	-	4.62 ± 0.15
14	2-Ethylhexan-1-ol	1035	-	1.36 ± 0.00	-	0.83 ± 0.33
15	Benzyl alcohol	1041	2.07 ± 0.19	57.92 ± 0.84	2.59 ± 0.23	52.48 ± 0.44
16	(*E*)-Oct-2-enal	1064	1.73 ± 0.24		0.86 ± 0.30	1.15 ± 0.09
17	Acetophenone	1073	1.41 ± 0.11	0.87 ± 0.08	1.23 ± 0.02	-
18	(*E*,*E*)-Octa-3,5-dien-2-one	1076	2.79 ± 0.17	1.41 ± 0.19	2.50 ± 0.16	1.46 ± 0.05
19	Nonanal	1108	-	4.43 ± 0.30	-	2.72 ± 0.47
20	2,6-Dimethylcyclohexan-1-ol	1114	-	-	-	1.62 ± 0.16
21	6-[(1*Z*)-Butenyl]-1,4-cycloheptadiene] (Dictyopterene D′)	1158	7.78 ± 0.48	1.81 ± 0.00	7.12 ± 0.20	1.41 ± 0.14
22	(*Z*)-Non-2-enal	1169	3.93 ± 0.24	-	4.45 ± 0.27	-
23	[6-Butyl-1,4-cycloheptadiene] (Dictyopterene C′)	1175	8.65 ± 0.78	-	9.34 ± 1.16	-
24	Decanal	1204	2.02 ± 0.20	1.46 ± 0.00	2.02 ± 0.18	1.03 ± 0.09
25	2-Phenylbut-2-enal (2-Phenylcrotonaldehyde)	1278	24.20 ± 1.36	-	21.72 ± 0.69	-
26	Heptadecane	1703	-	3.25 ± 0.20	-	2.32 ± 0.10
27	(*E*)-Nonadec-9-ene	1878	5.89 ± 0.83	2.90 ± 0.08	1.54 ± 0.32	2.60 ± 0.31

**Table 2:** The VOCs from *D. vermicularis* isolated by hydrodistillation (HD) and analysed by gas chromatography–mass spectrometry (GC–MS): (V—hydrodistillate of fresh *D. vermicularis*, VI—hydrodistillate of air-dried *D. vermicularis*).

No.	Compound	RI	Area% ± SD	
			V	VI
1	Nonane	900	0.36 ± 0.15	0.06 ± 0.01
2	Heptanal	904	-	0.12 ± 0.03
3	Benzaldehyde	968	0.08 ± 0.00	0.33 ± 0.12
4	Oct-1-en-3-ol	984	0.24 ± 0.05	0.22 ± 0.02
5	2-Pentylfuran	994	0.10 ± 0.01	0.22 ± 0.06
6	Octanal	1006	-	0.09 ± 0.03
7	(*E*,*E*)-Hepta-2,4-dienal	1015	-	0.06 ± 0.00
8	Benzyl alcohol	1040	-	0.15 ± 0.02
9	Phenylacetaldehyde	1051	0.15 ± 0.02	0.08 ± 0.00
10	(*E*)-Oct-2-enal	1064	-	0.08 ± 0.03
11	(*E*)-Oct-2-en-1-ol	1073	0.22 ± 0.07	0.24 ± 0.05
12	(*E*,*E*)-Octa-3,5-dien-2-one	1076	0.48 ± 0.23	0.16 ± 0.02
13	Nonan-2-one	1096	0.77 ± 0.17	0.03 ± 0.00
14	Linalool	1103	0.11 ± 0.00	-
15	Nonanal	1107	-	0.08 ± 0.01
16	2,6-Dimethylcyclohexan-1-ol	1113	-	0.40 ± 0.13
17	6-[(1*Z*)-Butenyl]-cyclohepta-1,4-diene] (Dictyopterene D′)	1158	0.17 ± 0.02	-
18	(*Z*)-Non-2-enal	1165	-	0.07 ± 0.02
19	[6-Butylcyclohepta-1,4-diene] (Dictyopterene C′)	1174	0.13 ± 0.03	-
20	2,4-Dimethylbenzaldehyde	1177	0.15 ± 0.01	-
21	Decan-2-one	1192	0.26 ± 0.07	-
22	Decanal	1204	3.66 ± 0.25	0.07 ± 0.01
23	(*Z*,*E*)-Nona-2,4-dienal	1218	-	0.06 ± 0.00
24	β-Cyclocitral	1226	-	0.08 ± 0.03
25	Decan-1-ol	1277	-	0.14 ± 0.04
26	2-Phenylbut-2-enal	1278	0.17 ± 0.06	-
27	2,6,11-Trimethyldodecane	1283	0.25 ± 0.04	-
28	Indole	1296	-	0.28 ± 0.09
29	(*Z*)-Tridec-3-ene	1296	0.46 ± 0.11	-
30	Undecanal	1311	-	0.09 ± 0.01
31	(*E*,*E*)-Deca-2,4-dienal	1320	-	0.26 ± 0.10
32	(*E*)-Undec-2-en-1-ol	1347	-	0.12 ± 0.03
33	β-Cubebene	1394	0.15 ± 0.06	
34	β-Elemene	1395	0.54 ± 0.10	0.18 ± 0.06
35	Dodecanal	1413	0.08 ± 0.01	0.13 ± 0.03
36	α-Ionone	1433	0.10 ± 0.01	0.12 ± 0.01
37	(*Z*)-Geranylacetone	1458	-	0.18 ± 0.04
38	(*E*)-Dodec-5-en-1-ol	1465	-	0.18 ± 0.03
39	Dodecan-1-ol	1479	1.18 ± 0.21	0.51 ± 0.15
40	Germacrene D	1485	0.72 ± 0.17	-
41	β-Ionone	1490	0.27 ± 0.11	2.61 ± 0.30
42	Pentadec-1-ene	1495	1.04 ± 0.17	0.29 ± 0.02
43	(*E*)-β-Guaiene	1498	0.76 ± 0.30	-
44	Tridecan-2-one	1499	-	0.31 ± 0.10
45	Pentadecane	1500	0.75 ± 0.22	0.27 ± 0.09
46	Germacrene A	1509	2.31 ± 0.31	0.20 ± 0.05
47	Tridecanal	1514	1.01 ± 0.18	0.23 ± 0.03
48	β-Cadinene	1520	1.31 ± 0.12	0.26 ± 0.02
49	Myristicine	1527	2.03 ± 0.32	-
50	Zonarene	1530	0.27 ± 0.03	-
51	(*E*)-Cadina-1,4-diene	1537	0.31 ± 0.07	-
52	Tridecan-1-ol	1581	1.25 ± 0.20	0.50 ± 0.05
53	Gleenol	1590	0.11 ± 0.00	-
54	Hexadecane	1600	0.21 ± 0.03	-
55	Tetradecanal	1616	0.33 ± 0.02	0.37 ± 0.03
56	Cubenol	1648	3.46 ± 0.56	0.65 ± 0.10
57	α-Cadinol	1660	0.47 ± 0.19	0.22 ± 0.02
58	Tetradecan-1-ol	1682	1.95 ± 0.20	3.16 ± 0.33
59	(*E*)-Heptadec-8-ene	1697	1.39 ± 0.31	0.37 ± 0.12
60	Heptadecane	1700	1.41 ± 0.10	1.32 ± 0.20
61	Pentadecanal	1719	2.02 ± 0.08	0.74 ± 0.08
62	Tetradecanoic acid	1770	1.86 ± 0.33	-
63	Octadec-1-ene	1780	0.15 ± 0.06	0.20 ± 0.02
64	Pentadecan-1-ol	1784	0.54 ± 0.09	1.64 ± 0.34
65	Octadecane	1800	-	0.10 ± 0.00
66	Hexadecanal	1821	0.57 ± 0.11	1.25 ± 0.25
67	6,10,14-Trimethylpentadecan-2-one	1850	0.56 ± 0.21	2.14 ± 0.36
68	(*Z*)-Hexadeca-1,9-diene	1865	0.42 ± 0.07	5.26 ± 0.44
69	Diisobutyl phthalate	1873	0.66 ± 0.08	1.66 ± 0.27
70	(*E*)-Nonadec-9-ene	1878	5.77 ± 0.61	12.79 ± 0.82
71	Hexadecan-1-ol	1885	1.42 ± 0.30	10.37 ± 0.36
72	Nonadec-1-ene	1897	0.36 ± 0.06	0.28 ± 0.07
73	Nonadecane	1900	0.85 ± 0.11	2.06 ± 0.34
74	Heptadecan-2-one	1911	0.19 ± 0.05	0.34 ± 0.10
75	(*E*,*E*)-Farnesyl acetone	1923	-	0.56 ± 0.11
76	Isophytol	1953	-	0.46 ± 0.10
77	Dibutyl phtalate	1967	-	0.57 ± 0.13
78	Hexadecanoic acid	1970	1.94 ± 0.26	0.76 ± 0.14
79	(*Z*)-Octadec-9-enal	1998	0.43 ± 0.10	0.28 ± 0.06
80	Eicosane	2000	0.09 ± 0.00	0.14 ± 0.04
81	Octadecanal	2024	0.59 ± 0.23	0.89 ± 0.24
82	Geranyllinalool	2033	-	0.16 ± 0.06
83	(*Z*)-Falcarinol	2045	2.52 ± 0.44	2.02 ± 0.59
84	Methyl heptadeca-5-8-11-trienoate	2049	0.91 ± 0.18	0.59 ± 0.18
85	(*Z*,*Z*,*Z*)-Octadeca-9,12,15-trien-1-ol	2056	0.36 ± 0.16	0.42 ± 0.08
86	(*Z*)-Octadec-9-en-1-ol	2061	0.20 ± 0.07	8.13 ± 0.96
87	(*Z*,*Z*)-Octadeca-3,13-dien-1-ol	2070	0.23 ± 0.03	-
88	Heneicos-10-ene	2075	0.83 ± 0.15	2.42 ± 0.42
89	Octadecan-1-ol	2088	0.78 ± 0.32	0.62 ± 0.11
90	Heneicosane	2100	0.36 ± 0.14	0.46 ± 0.02
91	(*Z*,*Z*)-Octadeca-9,12-dienoic acid	2110	1.34 ± 0.35	0.40 ± 0.15
92	(*E*)-Phytol	2116	16.69 ± 0.65	16.32 ± 1.01
93	Docosane	2200	7.69 ± 0.90	0.33 ± 0.14
94	(*E*)-Geranylgeraniol	2206	0.45 ± 0.12	-
95	Cembra-4,7,11,15-tetraen-3-ol	2231	4.30 ± 0.20	0.90 ± 0.20

**Table 3:** Major nonvolatile compounds in F3 and F4 fractions and their plausible identification by UHPLC-ESI(+)–HRMS.

							F3	F4
No.	t_R_ (min)	Name	Structure	Mono-Isotopic Mass	[M + H]^+^	Mass Difference (ppm)	Area (Counts)	
**Pigments**								
12	14.96	Fucoxanthin	C_42_H_58_O_6_	658.423340	659.43062	3.5	388,447	11,204
15	15.57	Pheophorbide *a*	C_35_H_36_N_4_O_5_	592.268570	593.27585	0.5	14,419	5581
19	16.68	Zeaxanthin/Lutein	C_40_H_56_O_2_	568.428040	569.43531	6.5	463,265	309,962
23	17.52	Siphonein	C_52_H_76_O_5_	780.56928	781.57655	5.2	97,166	131,660
28	20.04	Methyl (3*R*,10*Z*,14*Z*,20*Z*,22*S*,23*S*)-12-ethyl-3-hydroxy-13,18,22,27-tetramethyl-5-oxo-23-(3-oxo-3-{[(2*E*,7*R*,11*R*)-3,7,11,15-tetramethyl-2-hexadecen-1-yl]oxy}propyl)-17-vinyl-4-oxa-8,24,25,26-tetraazahexacycl;o [19.2.1.16,9.111,14.116,19.02,7]heptacosa-1(24),2(7),6(27),8,10,12,14,16,18,20-decaene-3-carboxylate	C_55_H_74_N_4_O_7_	902.555725	903.56303	0.4	69,855	2,573,134
29	20.05	3-Phorbinepropanoic acid, 9-acetyl-14-ethylidene-13,14-dihydro-21-(methoxycarbonyl)-4,8,13,18-tetramethyl-20-oxo-, 3,7,11,15-tetramethyl-2-hexadecen-1-yl ester	C_55_H_74_N_4_O_6_	886.560852	887.56811	2.0	677,361	38,222,272
30	20.18	Pheophytin *a*	C_55_H_74_N_4_O_5_	870.565918	871.5732	1.4	25,152	738,479
**Fatty Acid Derivatives**								
6	13.96	Palmitamide	C_16_H_33_NO	255.25621	256.26349	−2.0	243,821	3,675,708
7	13.99	1,3-Dihydroxy-2-propanyl 5,8,11,14-icosatetraenoate	C_23_H_38_O_4_	378.277008	379.28429	3.0	16,874	-
8	14.23	2,3-Dihydroxypropyl palmitate	C_19_H_38_O_4_	330.277008	331.28429	−5.2	110,342	253,278
9	14.35	Oleamide	C_18_H_35_NO	281.271851	282.27914	0.9	3,107,219	30,659,656
10	14.57	2,3-Dihydroxypropyl 9-octadecenoate	C_21_H_40_O_4_	356.292664	357.29994	2.0	19,415	-
13	15.30	2,3-Dihydroxypropyl stearate	C_21_H_42_O_4_	358.308319	359.31559	2.9	221,566	518,492
16	16.19	Erucamide	C_22_H_43_NO	337.334473	338.34174	2.4	670,677	2,888,407
17	16.38	2-Hydroxypropyl stearate	C_21_H_42_O_3_	342.313385	343.32067	2.1	-	188,262
18	16.48	3-{[6-O-(α-D-Galactopyranosyl)-β-D-galactopyranosyl]oxy}-2-[(*Z*)-9-hexadec-9-enoyloxy]propyl (*Z*,*Z*,*Z*)-9,12,15-octadecatrienoate	C_49_H_84_O_15_	912.580994	913.58830	−0.3	67,693	16,012
20	16.94	1-Hexadecanoyl-2-(9*Z*,12*Z*,15*Z*-octadecatrienoyl)-3-O-(α-D-galactosyl-1-6-β-D-galactosyl)-sn-glycerol	C_49_H_86_O_15_	914.596672	915.60395	0.3	161,165	68,963
22	17.38	*N*-(2-hydroxynonadecanoyl)-1-O-β-D-glucosyl-15-methylhexadecasphing-4-enine	C_42_H_81_NO_9_	743.59113	744.59841	−2.0	1,591,978	59,570,336
25	17.70	*N*-Nonadecanoyl-1-O-β-D-glucosyl-4-hydroxy-15-methylhexadecasphinganine	C_42_H_83_NO_9_	745.60678	746.61406	2.3	207,250	106,970,216
27	19.70	3-Hydroxy-1,2-propanediyl bis(9-octadecenoate)	C_39_H_72_O_5_	620.537964	621.54525	3.6	23,062	-
**Steroids and Terpenes**								
1	6.50	Loliolide	C_11_H_16_O_3_	196.10994	197.11722	1.9	44,368	4657
2	9.30	(3*S*,8*S*,9*S*,10*R*,13*S*,14*S*,17*R*)-17-[(2*E*)-4-Hydroxy-2-buten-2-yl]-10,13-dimethyl-2,3,4,7,8,9,10,11,12,13,14,15,16,17-tetradecahydro-1H-cyclopenta[a]phenanthren-3-ol	C_23_H_36_O_2_	344.271515	345.27881	4.9	575,025	283,200
3	9.44	(22*E*)-Chola-5,22-dien-3-ol	C_24_H_38_O	342.292267	343.29954	2.6	748,132	268,543
4	11.91	Sargaquinoic acid	C_27_H_36_O_4_	424.261353	425.26864	−3.5	5778	-
5	12.69	1′H-5α-Cholest-2-eno [3,2-b]indole	C_33_H_49_N	459.38650	460.39378	3.1	714,421	5,802,712
11	14.94	(3a*R*,4a*R*,6*S*,8a*S*)-1-Isopropyl-3a,8a-dimethyl-5-methylene-2,3,3a,4,4a,5,6,7,8,8a-decahydrobenzo[f]azulene-4a,6-diol	C_20_H_30_O_2_	302.22458	303.23186	5.6	146,533	3685
14	15.57	(3a*R*,4a*R*,6*S*,8a*R*)-1-Isopropyl-3a,8a-dimethyl-5-methylene-2,3a,4,5,6,7,8,8a,9,10-decahydrobenzo[f]azulene-4a,6(3H)-diol (Isoamijiol)	C_20_H_32_O_2_	304.240234	305.24751	2.7	173,463	16,323
21	17.02	11-Hydroxy-3,20-dioxopregn-4-en-21-yl (9*E*)-9-octadecenoate	C_39_H_62_O_5_	610.459717	611.4670	−2.4	129,163	22,853
24	17.57	6β-Hydroxystigmast-4-en-3-one	C_29_H_48_O_2_	428.36543	429.37271	1.5	504,060	230,167
26	18.44	(3β)-3-Hydroxystigmast-5-en-7-one	C_29_H_48_O_2_	428.365431	429.37271	−4.9	-	68,565

**Table 4:** Dose–inhibition results for *D. vermicularis* nonpolar fractions using ABTS in vitro assay (*n* = 4) to obtain the half-maximal inhibitory concentration (IC_50_) with presented confidence intervals, Hillslope, and R^2^ value.

Sample	IC_50_ Value (mg/mL)	Confidence Interval	Hillslope	R^2^ Value
F3	0.498	0.419–0.595	2.48	0.981
F4	0.798	0.696–0.937	1.97	0.992

## Data Availability

Data are contained within the article.
